# Design of limited-stop service based on the degree of unbalance of passenger demand

**DOI:** 10.1371/journal.pone.0193855

**Published:** 2018-03-05

**Authors:** Hu Zhang, Shuzhi Zhao, Huasheng Liu, Shidong Liang

**Affiliations:** 1 College of Transportation, Jilin University, Changchun, China; 2 Business School, University of Shanghai for Science and Technology, Shanghai, China; Beihang University, CHINA

## Abstract

This paper presents a limited-stop service for a bus fleet to meet the unbalanced demand of passengers on a bus route and to improve the transit service of the bus route. This strategy includes two parts: a degree assessment of unbalanced passenger demand and an optimization of the limited-stop service. The degree assessment of unbalanced passenger demand, which is based on the different passenger demand between stations and the unbalance of passengers within the station, is used to judge whether implementing the limited-stop service is necessary for a bus route. The optimization of limited-stop service considers the influence of stop skipping action and bus capacity on the left-over passengers to determine the proper skipping stations for the bus fleet serving the entire route by minimizing both the waiting time and in-vehicle time of passengers and the running time of vehicles. A solution algorithm based on genetic algorithm is also presented to evaluate the degree of unbalanced passenger demand and optimize the limited-stop scheme. Then, the proper strategy is tested on a bus route in Changchun city of China. The threshold of degree assessment of unbalanced passenger demand can be calibrated and adapted to different passenger demands.

## Introduction

With the expansion of cities, an effective transit system become increasingly important for sustainable land use planning under the influence of the travel demand [1]. The bus route has been extended and many stations are on the bus route. Due to the unbalance of passenger demand along the bus route, overload and inadequate utilization of bus space constantly appear. This is caused by the unified frequency of bus serving for each station on the entire bus route and severely affects the transit service and satisfaction of passengers. Although an increase of the priori frequency is widely adopted to meet the high passenger demand, the serving resource of buses would be wasted at stations with low demand and the number of buses within the bus fleet is limited. Thus, the unbalance of passenger demand should be considered and it is necessary to judge whether a traditional service is suitable for the unbalanced patterns of passengers. Buses should serve the segment with high demand intensively, while stations with low demand can be skipped to balance the passenger demand and improve transit service.

Previous studies presented many operational strategies to deal with the different demand patterns and the unbalanced distribution of passengers along the bus route [2–3]. Tétreault and El-Geneidy [4] considered the boarding and alighting behavior of passengers and the skipped action with limited-stop service to estimate the running time of buses. Their limited-stop service consists of a normal line and a limited-stop line. Leiva et al. [5] minimized the total cost based on waiting time, travel time, and operators’ costs to optimize the limited-stop service and to determine their frequencies by a given origin–destination matrix. Freyss et al. [6] implemented a continuous approximation model to determine the skipping stations based on a regular station density and affluence. Chiraphadhanakul and Barnhart [7] optimized a limited-stop line which runs parallel to the normal line by maximizing the benefit, i.e., the total in-vehicle time savings minus total increase in waiting time. Chen et al. [8] considered vehicle capacity and stochastic travel times and optimized the stopping design of a limited-stop bus service.

Moreover, short turning and deadheading strategies have also been proposed to concentrate on segments with high demand of two directions and to decrease the running time of buses between low-demand stations. Furth [9] and Ceder [10] suggested that the frequency of short-turn line is equal to the parameter *n* multiplied by the frequency of the all-stop line. Cortés et al. [11] implemented an integrated deadheading and short-turn strategy with a given OD matrix and by minimizing the total cost of user and operator. Liu et al. [12] considered the influence of random travel time and improved the reliability of the transit system with stop-skipping service.

In addition, several real-time control strategies have been studied using GPS data and smart card data [13–16]. Bin et al. [17] evaluated the reliability of the transit service to determine whether implementing the proposed partway deadheading strategy is meaningful. Bie et al. [18] developed a new algorithm based on GPS data to partition bus operating hours into time of day intervals. Yu et al. [19] used the variation of the intervals between a vehicle and its following vehicle to estimate the irregularity of transit operation and consequently presented a dynamic extra buses scheduling strategy.

These previous studies mainly addressed the optimal scheme in limited-stop service or other stop-skipping strategy and provide appropriate skipping stations via OD with unbalanced demand. There are few studies that evaluate the degree of unbalanced passenger demand and judge which demand pattern is required to implement the stop-skipping strategy. This paper focuses on the degree assessment of unbalanced passenger demand for an origin–destination matrix and optimization of a limited-stop service for a bus fleet. The degree assessment of unbalanced passenger demand considers both the different passenger demand between stations and the unbalance of passengers within each station. Variances of both parts determine the degree of unbalanced passenger demand for the bus route, which is used to judge whether a limited-stop service should be implemented. The threshold is calibrated via numerical test. If the degree of unbalanced passenger demand is high, the limited-stop service is optimized when considering the capacity of buses and allows buses to skip several proper skipping stations. Caused by stop skipping action and bus capacity, the waiting passengers who cannot board the buses are classified into four cases: (1) left over due to bus capacity, (2) left over due to skipped action, (3) left over due to bus capacity and skipped action, and (4) left over of the last skipped bus in the bus fleet. The proper skipping stations are determined by minimizing the total cost of the transit system, including the waiting time and in-vehicle time of passengers and the running time of buses. Following this introduction, Part 2 describes the problem setting for the unbalanced passenger demand. Part 3 evaluates the degree of unbalanced passenger demand. The optimization model for the limited-stop service is established and presented in Part 4 and Part 5 presents a solution method. Part 6 analyzes the results of our numerical test and the conclusions are presented in Part 7.

## Problem setting

**Fig 1:**
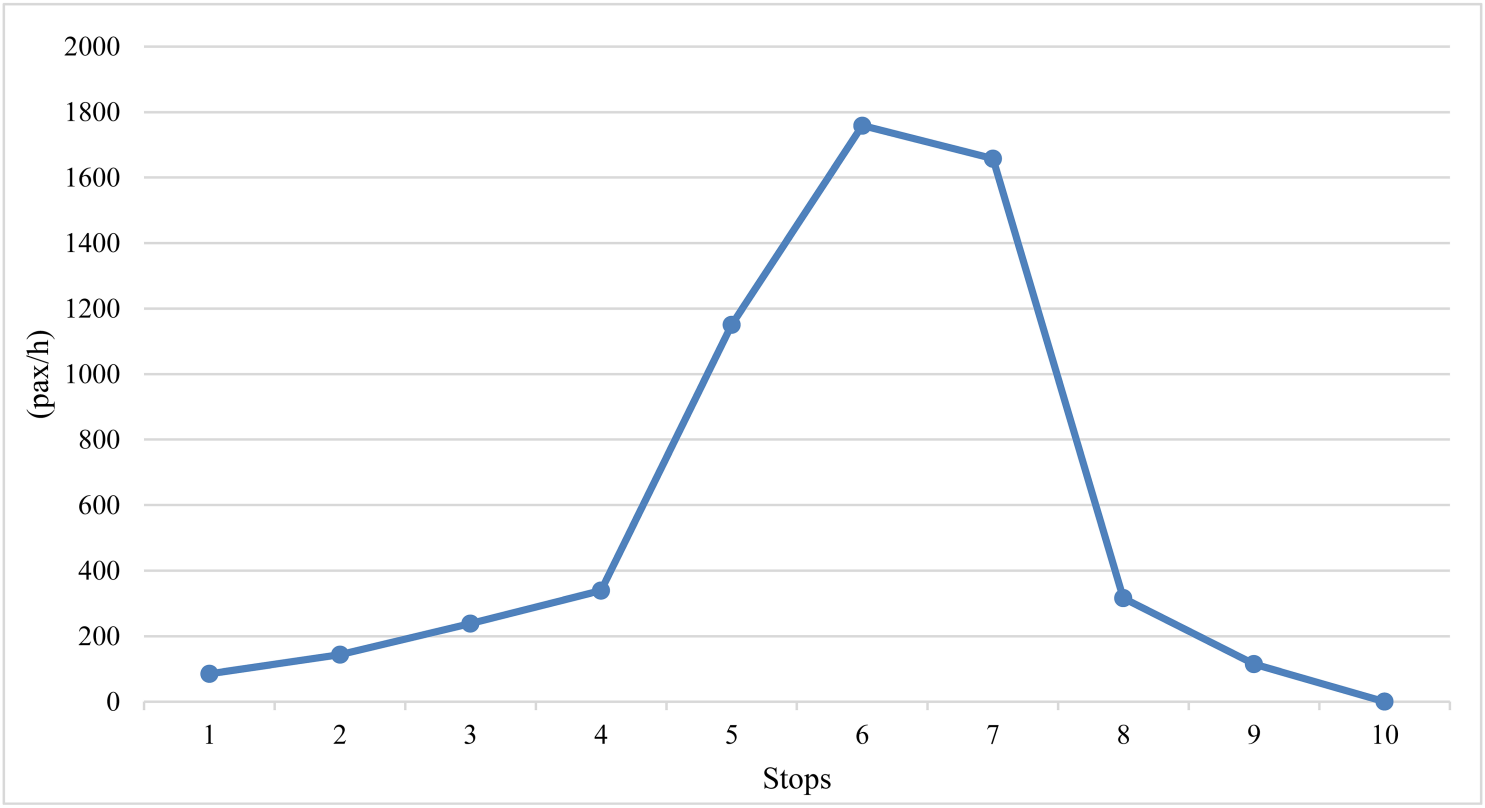
Load profile of a bus corridor.

**Fig 2:**
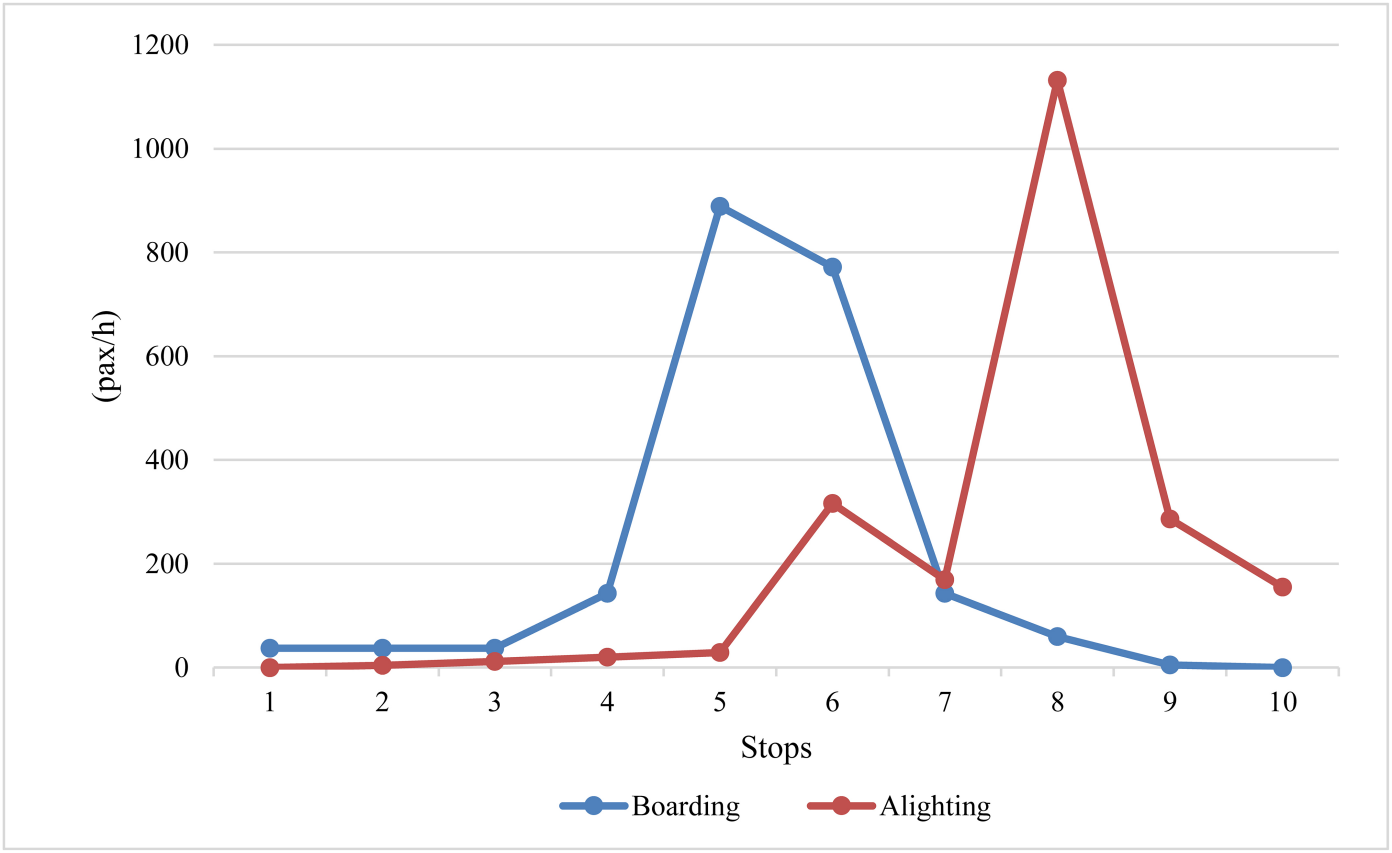
Passenger boarding and alighting demand.

The limited-stop service can be implemented based on an unbalance of passenger demand and such an unbalance of passenger demand exists in most bus routes. Figs 1 and 2 show a typical load profile for passenger boarding and alighting demand on a bus corridor with 10 stations [20]. It can be seen that the load of buses is different in the interval between consecutive stations and the boarding and alighting demand of each station are also unbalanced. Furthermore, the segment of unbalanced load is not identical to the boarding and alighting demand. The high demand of the load is concentrated on the middle of the bus line, between stations 5 and 7, while there is low boarding and alighting demand at station 7. This is because there is a high demand of passengers that want to board the bus from preceding stations and would pass station 7, compared to the demand of passenger boarding or alighting at station 7. This means that the unbalance of passenger demand is not only caused by the different patterns of boarding and alighting demand between stations, but also results from the origin–destination demand within each station. Therefore, the proposed degree assessment of the unbalanced passenger demand for the bus route should consider the different passenger demand between stations and the unbalance of passengers within the station.

A bus route with *N* stations is studied, operated by a bus fleet of size *m*. The index of vehicles is denoted as *i* and each station is denoted as *j*. *λ*_*l*,*j*_ represents the demand of passengers between station *l* and station *j*. With a traditional service, buses serve entire stations where passengers can board and alight. However, under limited-stop service, buses would skip several stations where there is no boarding and alighting.

## Degree assessment of unbalanced passenger demand

Unbalance of passenger demand results from the difference of origin–destination of passengers. Due to the environment around the stations, stations along the bus route gather passengers at different demand. At a station, passengers boarding a same bus will alight at different stations. In this paper, the degree assessment of unbalanced passenger demand will be determined from two aspects: the different passenger demand between stations and the unbalance of passengers within a station.

### Degree assessment of unbalanced passenger demand between stations

The limited-stop service allows buses to skip proper stations where passengers consequently cannot board or alight from the bus. Therefore, to save waiting time and avoid unnecessary transfer of passengers, the skipped stations should gather little passengers and there should be low alighting demand of passenger from preceding stations. Thus, passenger demand between stations includes both boarding and alighting demand at a station. The variance of passenger demand is used to estimate the degree of unbalanced passenger demand between stations.

$$\lambda_{k}^{+}=\sum_{j=k+1}^{N}\lambda_{l=k,j}$$ (1)

$$\lambda_{k}^{-}=\sum_{l=1}^{k-1}\lambda_{l,j=k}$$ (2)

$$\bar{p}=\frac{1}{N}\cdot \sum_{k=1}^{N}\left(\lambda_{k}^{+}+\lambda_{k}^{-}\right)$$ (3)

$$\sigma_{}^{Between}=\sqrt{\frac{\sum_{k=1}^{N}{\left(\lambda_{k}^{+}+\lambda_{k}^{-}-\bar{p}\right)}^{2}}{N-1}}$$ (4)

where $\lambda_{k}^{+}$ represents the boarding demand at station *k*. $\lambda_{k}^{-}$ represents the alighting demand at station *k*. $\lambda_{k}^{+}+\lambda_{k}^{-}$ describes the passenger demand at station *k*. *σ*^*Between*^ represents the variance of passenger demand, which presents the degree of unbalanced passenger demand between stations. Small values of this parameter indicate a high degree of balanced passenger demand, while large values indicate that passenger demand along the bus route is unevenly distributed.

### Degree assessment of unbalanced passenger demand within the station

At each station, the boarding passengers have different destinations. Skipping several stations for the high origin–destination demand of passengers can save in-vehicle time during their trips. Thus, the degree of unbalanced passenger demand within the station was also studied.

$${\bar{\lambda }}_{k}=\frac{1}{N-k}\cdot \sum_{j=k-1}^{N}\lambda_{l=k,j}$$ (5)

$$\sigma_{k}^{Within}=\sqrt{\frac{\sum_{j=k-1}^{N}{\left(\lambda_{l=k,j}-{\bar{\lambda }}_{k}\right)}^{2}}{N-k-1}}$$ (6)

where ${\bar{\lambda }}_{k}$ represents the mean passenger demand from station *k* to the subsequent stations within station *k*. $\sigma_{k}^{Within}$ represents the variance of passenger demand within the station.

### Determination for implementing limited-stop service

To comprehensively reflect the degree of unbalanced passenger demand of the transit system, both the variance of passenger demand between and within stations are considered. The degree of unbalanced passenger demand along the bus route can be defined as follows:

$$Ε=\sigma_{}^{Between}/\bar{p}+\sum_{k=1}^{N-1}\sigma_{k}^{Within}/{\bar{\lambda }}_{k}$$ (7)

If the degree of unbalanced passenger demand of the transit system is higher than a given threshold (E^*threshold*^), this indicates an uneven distribution along the bus route and a larger differentiated OD demand of passengers. Buses should intensively serve stations with high demand. For this condition, a limited-stop service will be implemented to save the cost of the transit system and to fully utilize the resources of the operator.

$$\text{E}\geq {\text{E}}^{threshold}$$ (8)

## Optimization model

### Assumptions and definition of variables

For the condition that the unbalanced passenger demand of the transit system is higher than the threshold and that the limited-stop service needs to be implemented, proper skipping stations in the limited-stop service should be determined. The limited-stop service can increase the speed of buses, save in-vehicle time, and decrease the waiting time of passengers at serving stations; however, it increases the waiting time of passengers at skipping stations because those passengers have to wait for the next bus. Therefore, to determine the appropriate skipping stations, the optimization of limited-stop service should integrate the cost of the waiting time and the in-vehicle time of passengers as well as the running time of buses. Whether Bus *i* serves Station *j* is denoted as *y*_*i*,*j*_ (*y*_*i*,*j*_ ∈ {0, 1}). If the bus stops at the station, *y*_*i*,*j*_ = 1; otherwise, *y*_*i*,*j*_ = 0. We assume that consecutive buses cannot skip the same stations to avoid a long waiting time for passengers, namely, *y*_*i*,*j*_+ *y*_*i-*1,*j*_ = 1. Fast buses and the last bus are not allowed to skip the same station, thus: *y*_1,*j*_+ *y*_*m*,*j*_ = 1.

The definition of variables used throughout the model formulation is as follows:

*m* Total number of buses in the bus fleet.
$A_{i,j}^{+}$ Number of arriving passengers during the interval between the bus *i* and *i*+1.
*h*_*i*,*j*_ Interval between bus *i* and *i*+1.
$T_{i,j}^{A}$ Arrival time of bus *i* at station *j*.
*λ*_*i*,*j*→*k*_ Arrival rate of passengers riding bus *i* from station *j* to station *k*.
$\Delta P_{i,j}^{+}$ Number of passenger left over by the bus capacity of bus *i*-1 to board the bus *i*.
$T_{i,j}^{W,\Delta P}$ Waiting time of passengers $\Delta P_{i,j}^{+}$.
$\Delta S_{i,j}^{+}$ Number of passenger left over by the skipped action of bus *i*-1 to board bus *i*.
$T_{i,j}^{W,\Delta S}$ Waiting time of passengers $\Delta S_{i,j}^{+}$.
$\Delta I_{i,j}^{+}$ Number of passengers left over due to bus capacity and skipped action to board the bus *i*.
$T_{i,j}^{W,\Delta I}$ Waiting time of passengers $\Delta I_{i,j}^{+}$.
$\Delta L_{m,j}^{+}$ Number of passengers left over due to the last skipped bus in the bus fleet.
$T_{m,j}^{W,\Delta L}$ Waiting time of passengers $\Delta L_{m,j}^{+}$.
*L*_*i*-1,*j*−1_ Load of bus *i*-1 departing from stop *j*-1.
$N_{i-1,j}^{-}$ Number of passengers alighting from bus *i*-1.
*N*_*c*_ Capacity of the bus.
*H* Average departure interval.
$N_{i,j}^{+}$ Number of passengers that can board the bus *i* at station *j*.
$T_{i,j}^{W}$ Waiting time of passengers for bus i at station j.
$T_{i,j}^{E}$ Dwelling time of bus *i* at station *j*.
*a* Boarding time per passenger.
*b* Alighting time per passenger.
$T_{i,j}^{IN}$ In-vehicle time of passengers between station *j* and station *j*+1.
$T_{i}^{R}$ Running time of bus *i* that completes the entire bus route.
$T_{i,j}^{D}$ Departing time of bus *i* from station *j*.
*C* Total cost of the transit system.
*C*_*W*_ Cost of the waiting time of passengers.
*C*_*IN*_ Cost of the in-vehicle time of passengers.
*C*_*R*_ Cost of running time of buses.

### Limited-stop service formulation

The cost of waiting time relates to the number of passengers and the average waiting time of passengers that can board buses. When a bus arrives at a station, the boarding passengers include passenger that arrived during the interval between serving buses and passengers left by the preceding bus.

The number of passengers during interval between bus *i* and *i*+1 can be expressed as:

$$A_{i,j}^{+}=h_{i,j}^{}\cdot \sum_{k=j+1}^{N}\lambda_{i,j\to k}^{}$$ (9)

$$h_{i,j}^{}=T_{i,j}^{A}-T_{i-1,j}^{A}$$ (10)

where $A_{i,j}^{+}$ represents the number of arrival passengers during the interval between the bus *i* and *i*+1, which is denoted as *h*_*i*,*j*_, at station *j*. $T_{i,j}^{A}$ represents the arrival time of bus *i* at station *j*.

**Fig 3:**
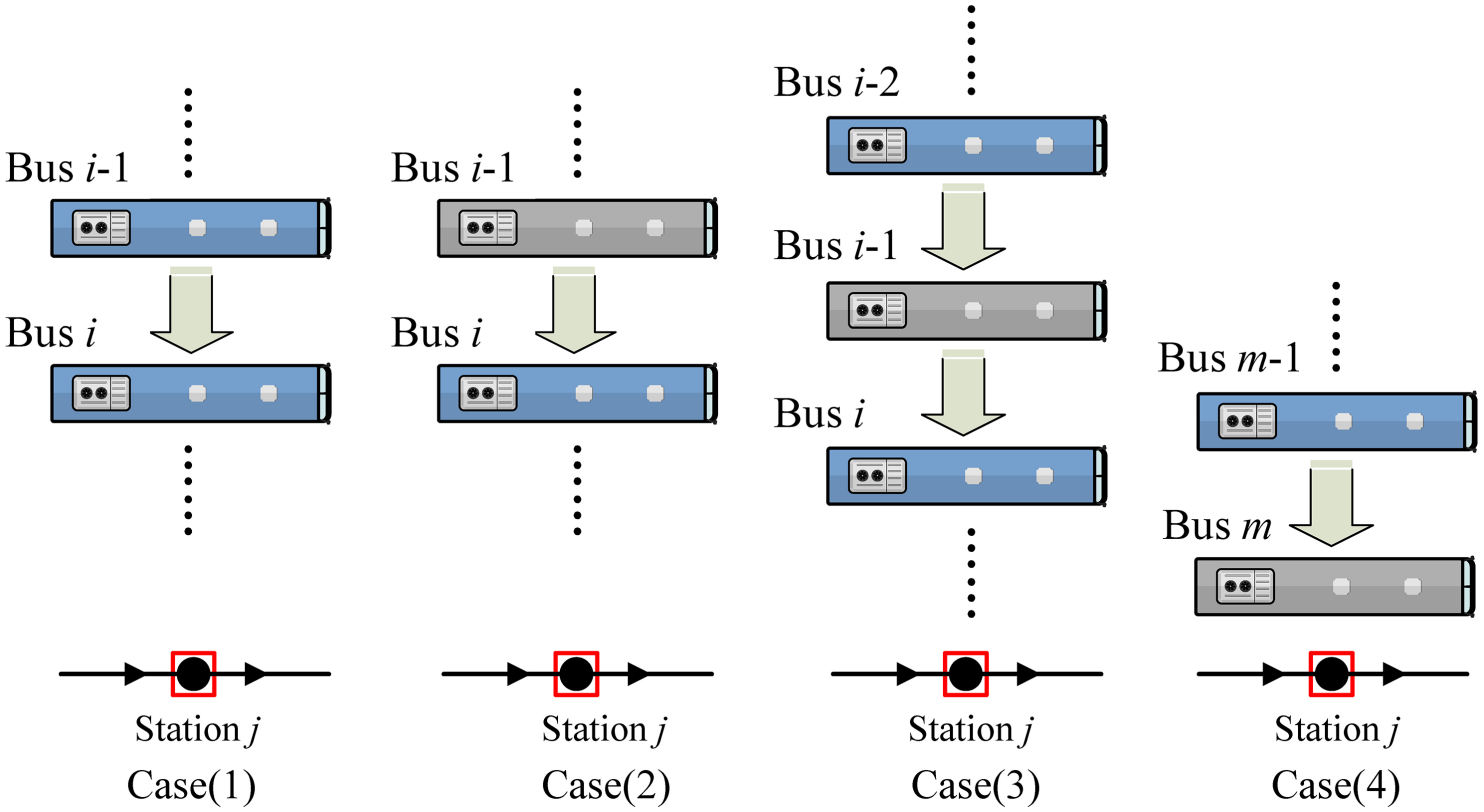
Classification of waiting passengers who cannot board the buses.

Under the influence of both stop skipping action and bus capacity, part of the waiting passengers may not be able to board the buses arriving at the station. This leaves passengers for the next serving bus if the skipping buses do not serve the station or if the number of waiting passengers exceeds the vehicle capacity. If the last bus skips several stations in the optimization scheme, the waiting passengers at those stations will be left for the next optimization scheme and the waiting time will be extended (other studies always ignore this part). In this paper, we classify the waiting passengers who cannot board the buses into four cases corresponding to Fig 3.

The number of waiting passengers and the waiting time for Case (1), Case (2), Case (3), and Case (4) will be explained and calculated as follows:

- Case (1): waiting passengers left over by the bus capacity

As shown in Fig 3, Case (1) describes that both the preceding bus *i*-1 and the bus *i* serve the station *j* and there are some passengers who cannot board the bus *i*-1 and consequently they have to wait for the bus *i* because the bus *i*-1 reached capacity. The number of left-over passenger due to the bus capacity of bus *i*-1 to board the bus *i* can be obtained by:

$$\Delta P_{i,j}^{+}=\{\begin{array}{ll} L_{i-1,j-1}+A_{i-1,j}^{+}-N_{i-1,j}^{-}-N_{c} & if\;L_{i-1,j-1}+W_{i-1,j}^{+}-N_{i-1,j}^{-}\geq N_{c}\;\cap y_{i-1,j}=y_{i,j}=1\\ 0 & otherwise \end{array}$$ (11)

$$L_{i,k}=\sum_{j=1}^{k}\left(N_{i,j}^{+}-N_{i,j}^{-}\right)$$ (12)

$$N_{i,j}^{-}=\sum_{k=1}^{j-1}N_{i,k}^{+}$$ (13)

where *L*_*i*-1,*j*−1_ represents the load of the bus *i*-1 departing from stop *j*-1. $A_{i-1,j}^{+}$ represents the number of passengers during the interval between bus *i*-1 and the preceding bus. $N_{i-1,j}^{-}$ represents the number of passengers alighting from bus *i*-1. *N*_*c*_ represents the capacity of the bus. $N_{i,k}^{+}$ represents the number of passengers that can board bus *i* at station *k*, which is determined by the remaining space in buses.

The waiting time of passengers $\Delta P_{i,j}^{+}$ can be computed by:

$$T_{i,j}^{W,\Delta P}=\{\begin{array}{ll} \Delta P_{i,j}^{+}\cdot (\frac{1}{2}h_{i-1,j}+h_{i,j}) & if\;L_{i-1,j-1}+W_{i-1,j}^{+}-N_{i-1,j}^{-}\geq N_{c}\;\cap y_{i-1,j}=y_{i,j}=1\\ 0 & otherwise \end{array}$$ (14)

- Case (2): the waiting passengers left over due to skipped action

In Case (2), bus *i*-1 skips the station *j* and bus *i* serves the station *j*. Arriving passengers during the interval between bus *i*-1 and *i*-2 cannot board the bus *i*-1. The number of passenger left over due to the skipped action of the bus *i*-1 to board the bus *i* can be obtained by:

$$\Delta S_{i,j}^{+}=\{\begin{array}{ll} A_{i-1,j}^{+} & if\;y_{i-1,j}=0\cap y_{i,j}=1\\ 0 & otherwise \end{array}$$ (15)

The waiting time of passengers $\Delta S_{i,j}^{+}$ can be obtained by:

$$T_{i,j}^{W,\Delta S}=\{\begin{array}{ll} \Delta S_{i,j}^{+}\cdot (\frac{1}{2}h_{i-1,j}+h_{i,j}) & if\;y_{i-1,j}=0\cap y_{i,j}=1\\ \begin{array}{ll} 0 & otherwise \end{array} & \end{array}$$ (16)

- Case (3): waiting passengers left over due to bus capacity and skipped action

Case (3) shows that the bus *i*-2 reaches capacity, which is similar to Case (1), and the bus *i*-1 skips the station *j*. Then, the passengers left over by the bus i-2 should wait for bus *i*. The number of passengers left over due to bus capacity and the skipped action to board the bus *i* can be obtained by:

$$\Delta I_{i,j}^{+}=\{\begin{array}{ll} L_{i-2,j-1}+A_{i-2,j}^{+}-N_{i-2,j}^{-}-N_{c} & if\;L_{i-2,j-1}+W_{i-2,j}^{+}-N_{i-2,j}^{-}\geq N_{c}\cap y_{i-2,j}=y_{i,j}=1\cap y_{i-1,j}=0\\ 0 & otherwise \end{array}$$ (17)

The waiting time of passengers $\Delta I_{i,j}^{+}$ can be obtained by:

$$T_{i,j}^{W,\Delta I}=\{\begin{array}{ll} \Delta I_{i,j}^{+}\cdot (\frac{1}{2}h_{i-2,j}+h_{i-1,j}+h_{i,j}) & \;if\;L_{i-2,j-1}+W_{i-2,j}^{+}-N_{i-2,j}^{-}\geq N_{c}\cap y_{i-2,j}=y_{i,j}=1\cap y_{i-1,j}=0\\ \begin{array}{ll} 0 & otherwise \end{array} & \end{array}$$ (18)

- Case (4): waiting passengers left over from the last skipped bus in the bus fleet

In this paper, the number of buses in the bus fleet is set as m. Waiting passengers who are skipped by the last bus *m* in the optimization scheme at those stations will be left to the next optimization scheme and this waiting time should be integrated into the optimization of the last bus *m*. The number of passengers left over from the last skipped bus in the bus fleet can be obtained by:

$$\Delta L_{m,j}^{+}=\{\begin{array}{ll} A_{m,j}^{+} & if\;i=m\cap y_{m,j}=0\\ 0 & otherwise \end{array}$$ (19)

The waiting time of passengers $\Delta L_{m,j}^{+}$ can be obtained by:

$$T_{m,j}^{W,\Delta L}=\{\begin{array}{ll} \Delta L_{m,j}^{+}\cdot (\frac{1}{2}h_{m,j}+H) & if\;i=m\cap y_{m,j}=0\\ \begin{array}{ll} 0 & otherwise \end{array} & \end{array}$$ (20)

where *H* is introduced to consider the waiting passengers left over from the last skipped bus *m* and is set as the average departure interval.

To sum up the above four cases, the number of passengers that can board the bus *i* at station *j* can be obtained via:

$$N_{i,j}^{+}=\{\begin{array}{ll} N_{c}-N_{i,j}^{-}+L_{i,j-1} & if\;L_{i,j-1}+W_{i,j}^{+}-N_{i,j}^{-}\geq N_{c}\\ \Delta P_{i,j}^{+}+\Delta S_{i,j}^{+}+\Delta I_{i,j}^{+}+A_{i,j}^{+}\cdot y_{i,j} & otherwise \end{array}$$ (21)

Then, in addition to the above four waiting times of the remaining passengers, the waiting time of passengers for bus *i* at station j should also include the waiting time of arriving passengers during the interval between bus *i* and *i*+1

$$T_{i,j}^{W}=T_{i,j}^{W,\Delta P}+T_{i,j}^{W,\Delta S}+T_{i,j}^{W,\Delta I}+T_{m,j}^{W,\Delta L}+A_{i,j}^{+}\cdot h_{i,j}^{}\cdot y_{i,j}$$ (22)

Regarding the cost of in-vehicle time, passengers spend their in-vehicle time on running time between stations and dwelling time at stations. The running time between stations can be obtained from historical data and the running time between station *j* and station *j*+1 can be denoted as $T_{j}^{R}$. The dwelling time depends on the behavior of boarding and alighting, which is the maximum value between boarding time and alighting time. The dwelling time of bus *i* at station *j* can be obtained as follows:

$$T_{i,j}^{E}=\{\begin{array}{ll} \text{max}[a\cdot N_{i,j}^{+},b\cdot N_{i,j}^{-}] & if\;y_{i,j}=1\\ 0 & if\;y_{i,j}=0 \end{array}$$ (23)

where *a* is the boarding time per passenger and *b* is the alighting time per passenger.

Thus, the in-vehicle time of passengers between station *j* and station *j*+1 can be obtained by:

$$T_{i,j}^{IN}=(T_{j}^{R}+y_{i,j}\cdot T_{i,j}^{E})\cdot L_{i,j}$$ (24)

The running time of buses includes the running time between stations and the dwelling time at stations, which is similar to the in-vehicle time of passengers. The running time of bus *i* that completes the entire bus route can be obtained by:

$$T_{i}^{R}=\sum_{j=1}^{N-1}\left(T_{i,j}^{A}-T_{i-1,j}^{A}\right)$$ (25)

$$T_{i,j}^{A}=T_{i,j-1}^{D}+T_{j-1}^{R}$$ (26)

$$T_{i,j}^{D}=T_{i,j}^{A}+T_{i,j}^{E}\cdot y_{i,j}$$ (27)

where $T_{i,j}^{D}$ is the departing time of bus *i* from station *j*.

The total cost of the transit system includes the cost of waiting time and in-vehicle time of passengers and the cost of the running time of buses. The object of the optimization model is to minimize the total cost of the transit system.

$$\begin{array}{ll} \text{min}C & =C_{W}+C_{IN}+C_{R}\\ & =\sum_{i}^{m}(c_{W}\cdot \sum_{j=1}^{N-1}T_{i,j}^{W}+c_{IN}\cdot \sum_{j=1}^{N-1}T_{i,j}^{IN}+c_{R}\cdot T_{i}^{R}) \end{array}$$ (28)

where *c*_*W*_, *c*_*IN*_, and *c*_*R*_ are values of waiting time, in-vehicle time, and running time, respectively.

## Solution methods

**Fig 4:**
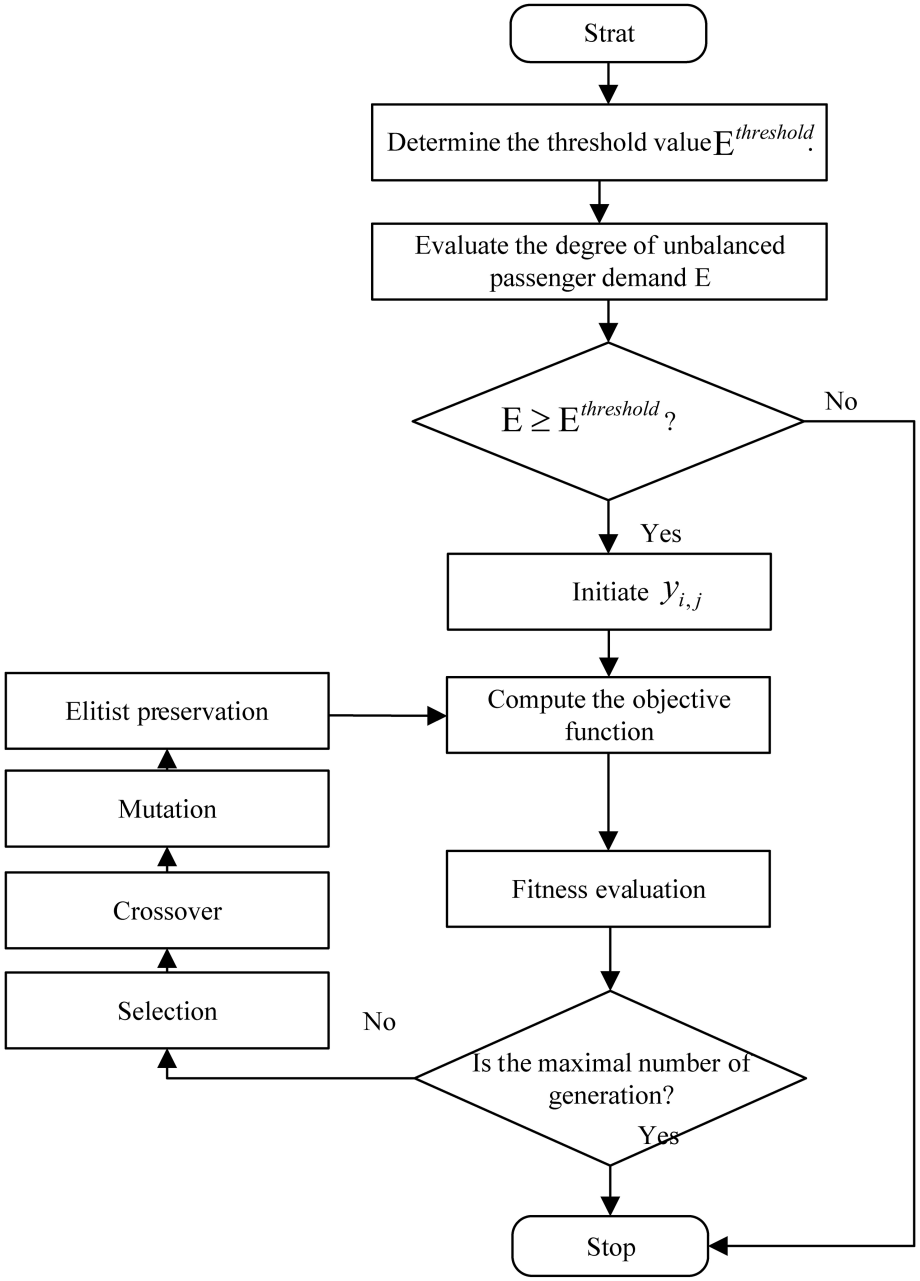
Solution algorithm.

To solve the objective function (22), a solution algorithm is presented that evaluates the degree of unbalanced passenger demand in the bus route and determines the proper skipped stations for the limited-stop service. The solution algorithm includes two parts that determine whether it is required to implement the limited-stop service and to output the optimization scheme. Considering that the design of the limited-stop service is a nonlinear problem [0, 1], the genetic algorithm is used to yield a minimum-cost transit operation to optimize transit routes [21–22]. Genetic algorithms can efficiently cope with mixed-integer non-linear problems and the objective function gradient does not require calculation, thus reducing computational effort. Fig 4 illustrates the solution algorithm for the proposed strategy. The specific steps of this solution method, corresponding to Fig 4, are as follows:

Step 1 Input the passenger demand (*λ*_*i*,*j*_) and the number of the entire bus fleet (*m*) and determine the threshold (E^*threshold*^);
Step 2 Evaluate the current degree of unbalanced passenger demand (E);
  Step 2.1 Compute the degree assessment of the unbalanced passenger demand between stations (*σ*^*Between*^), according to Eqs (1)–(4);
  Step 2.2 Evaluate the degree of unbalanced passenger demand within the station ($\sigma_{k}^{Within}$), according to Eqs (5) and (6);
  Step 2.3 Determine the degree of unbalanced passenger demand along the bus route (E), according to Eq (7). If E ≥ E^*threshold*^, go to Step 3; otherwise go to Step 5;
Step 3 Optimize the limited-stop strategy;
  Step 3.1 Initiate skipping schemes (*y*_*i*,*j*_); set the number of iterations for the genetic algorithm, the crossover probability *P*_*c*_, and the mutation probability *P*_*m*_. A chromosome consists of the entire bus fleet, as shown below:

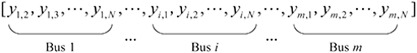

  Step 3.2 Calculate the value of the objective function (*C*), according to Eq (28). Because the objective function is formed to minimize benefits, the objective function is equal to the reciprocal of the fitness function;
  Step 3.3 The selection, crossover, and mutation of chromosomes are applied to produce new chromosomes. The crossover probability is set to 0.5, the mutation probability is set to 0.01;
  Step 3.4 An elitist preservation strategy is adopted to improve both search speed and search precision. A sample after Step 4 is replaced with the best sample before Step 3.3;
  Step 3.5 If the maximal number of generations is exceeded, then stop; Otherwise, go to Step 3.2;
Step 4 Output the optimization program;

## Numerical test

**Fig 5:**
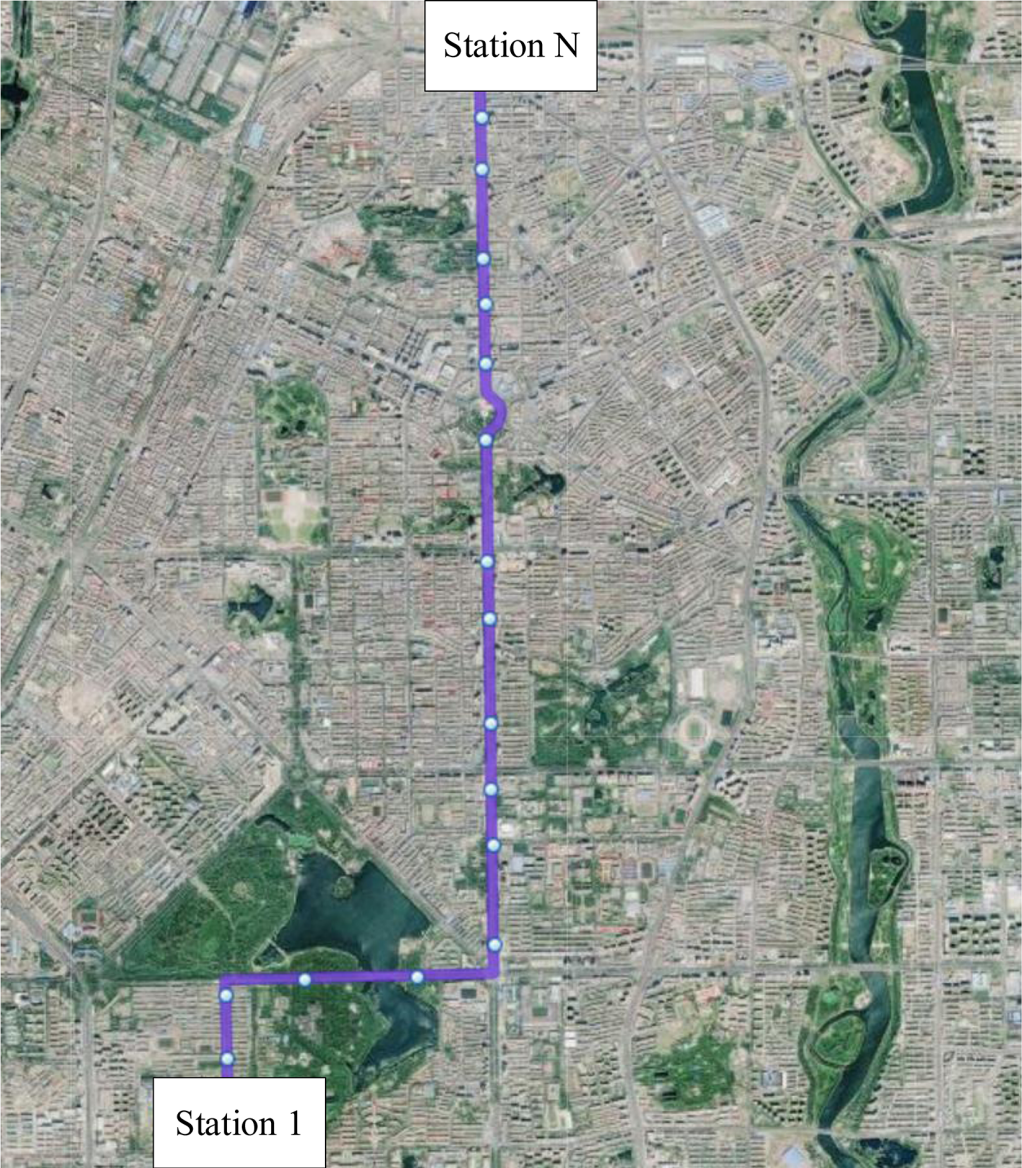
bus route number 6 of Changchun city in China.

The proposed strategy is tested on the bus route number 6 of Changchun City in China, as shown in Fig 5. Twenty stations are on the bus route with a length of 9.9 km. The origin of the bus route is mainly within residential areas and the destination is located downtown. The running time between the origin and destination of the bus route is about 28 min. The travel speed of buses is about 13 km/h. The average passenger boarding and alighting times are 1.5 sec and 3.0 sec, respectively, referring to the practicalities of reinvestigation and a previously published study (Chen et al. 2015). The time value is assumed as $15/pax-h for waiting time, $10/pax-h for in-vehicle time, and $50/veh-h for running time. The number of the entire bus fleet *m* is six vehicles.

**Fig 6:**
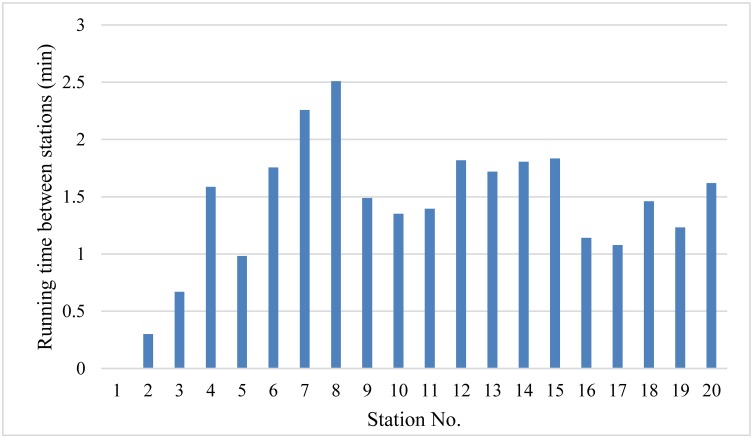
Running time between stations of bus route number 6.

**Fig 7:**
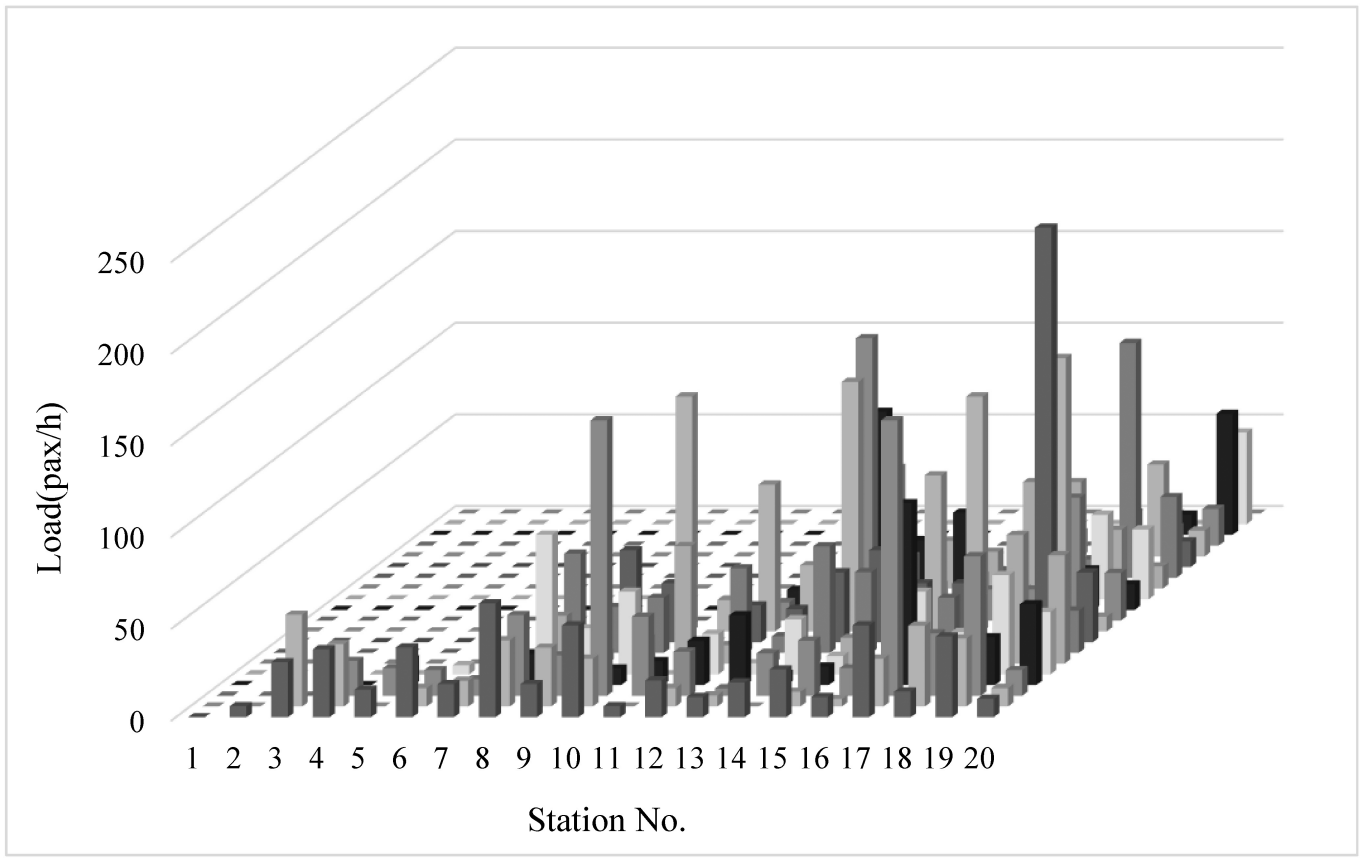
Average passenger demand during the peak period.

**Fig 8:**
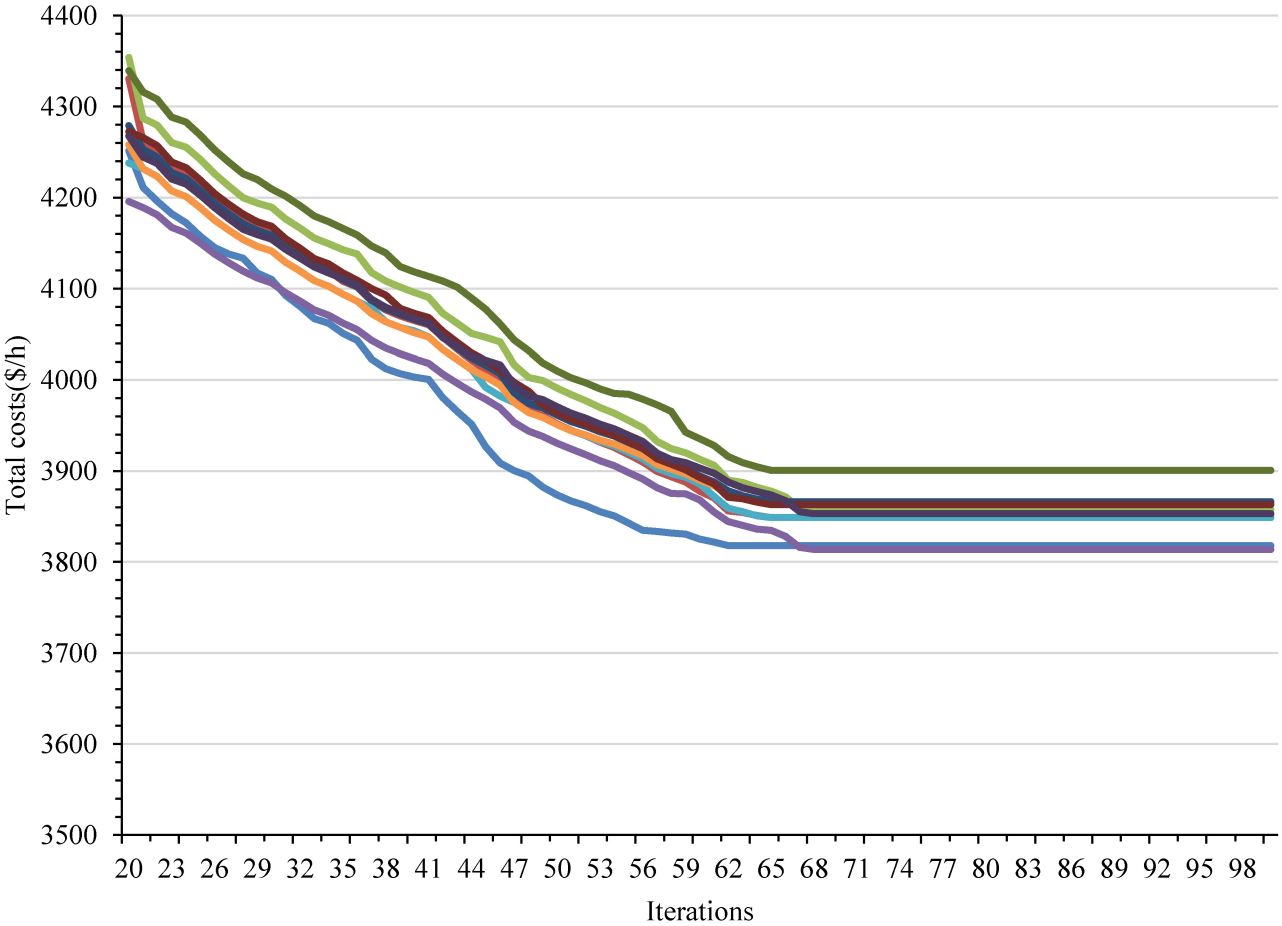
Result of each iteration of the genetic algorithm.

Historical data of passenger demand was collected during 30 days. We obtained the bus OD data, which records the boarding and alighting station of each passenger, and the running time between stations via on-board surveys of the entire bus route during the peak time period. Furthermore, the running time between stations of the bus route number 6 is shown in Fig 6. At this time, the average passenger demand shows an unbalanced distribution, as shown in Fig 7. In the solution method, the number of iterations of the genetic algorithm NG is set to 100. The crossover probability is set to 0.5, and the mutation probability is set to 0.01. Fig 8 depicts the convergence of the calculation with experimentation for 10 times. The algorithm has good convergence and optimal solutions of the objective function are found within 70 iterations. The mathematical software MATLAB R2011a is applied to simulate bus operations, which is expected to provide a fairly reliable environment to test the proposed strategy. Furthermore, the data is used to determine the threshold of degree of unbalanced passenger demand to judge which services are suitable for the bus route.

### Determination of the threshold of the degree of unbalanced passenger demand

The threshold of the degree of unbalanced passenger demand can reveal whether it is necessary to implement the limited-stop service. A high degree of unbalanced passenger demand indicates potential skipping stations which can save the total cost of the transit system. Test data is used to construct an examination. In this examination, the degree of unbalanced passenger demand is evaluated and the corresponding total costs of transit system with and without the limited-stop service are computed.

**Fig 9:**
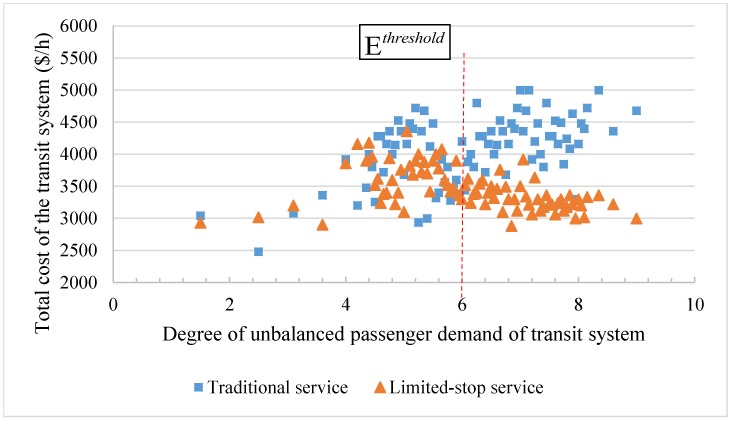
Total cost of the transit system under the different degree of unbalanced passenger demand.

Fig 9 shows the total cost of the transit system both with and without the limited-stop service under different degrees of unbalanced passenger demand. This shows that the degree of unbalanced passenger demand concentrates between stations 4 and 8 and the cost of the transit system with traditional service is random under different degrees of unbalanced passenger demand. Furthermore, when the degree of unbalanced passenger demand is below 6.0, the limited-stop service causes little difference to the traditional service. This is because the distribution of passenger demand is relatively flat. Skipping several stations has little influence on saving the total cost of the transit system and skipping stations are even completely removed through the optimization. However, when the degree of unbalanced passenger demand is higher than 6.0 (indicating that there are potential skipping stations), limited-stop service exerts a strong effect on saving the total cost. Therefore, the threshold of the degree of unbalanced passenger demand is set to 6.0.

### Results

**Fig 10:**
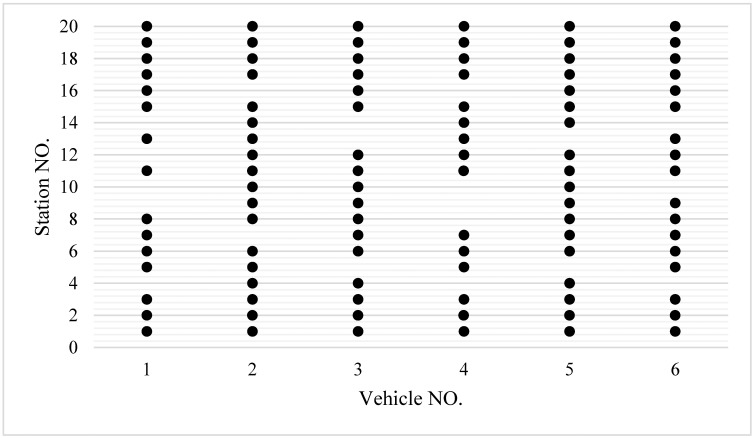
Proper skipping stations with limited-stop service.

To evaluate the performance of the proposed strategy, a dataset with a degree of unbalanced passenger demand of 6.3, is optimized to determine the skipping stations. Fig 10 shows the optimization scheme of a transit system for six vehicles. The segment with low demand of passengers (including boarding demand and alighting demand) concentrates on stations 4, 9, and 14. Each bus skips several stations and consecutive buses cannot skip the same station.

**Fig 11:**
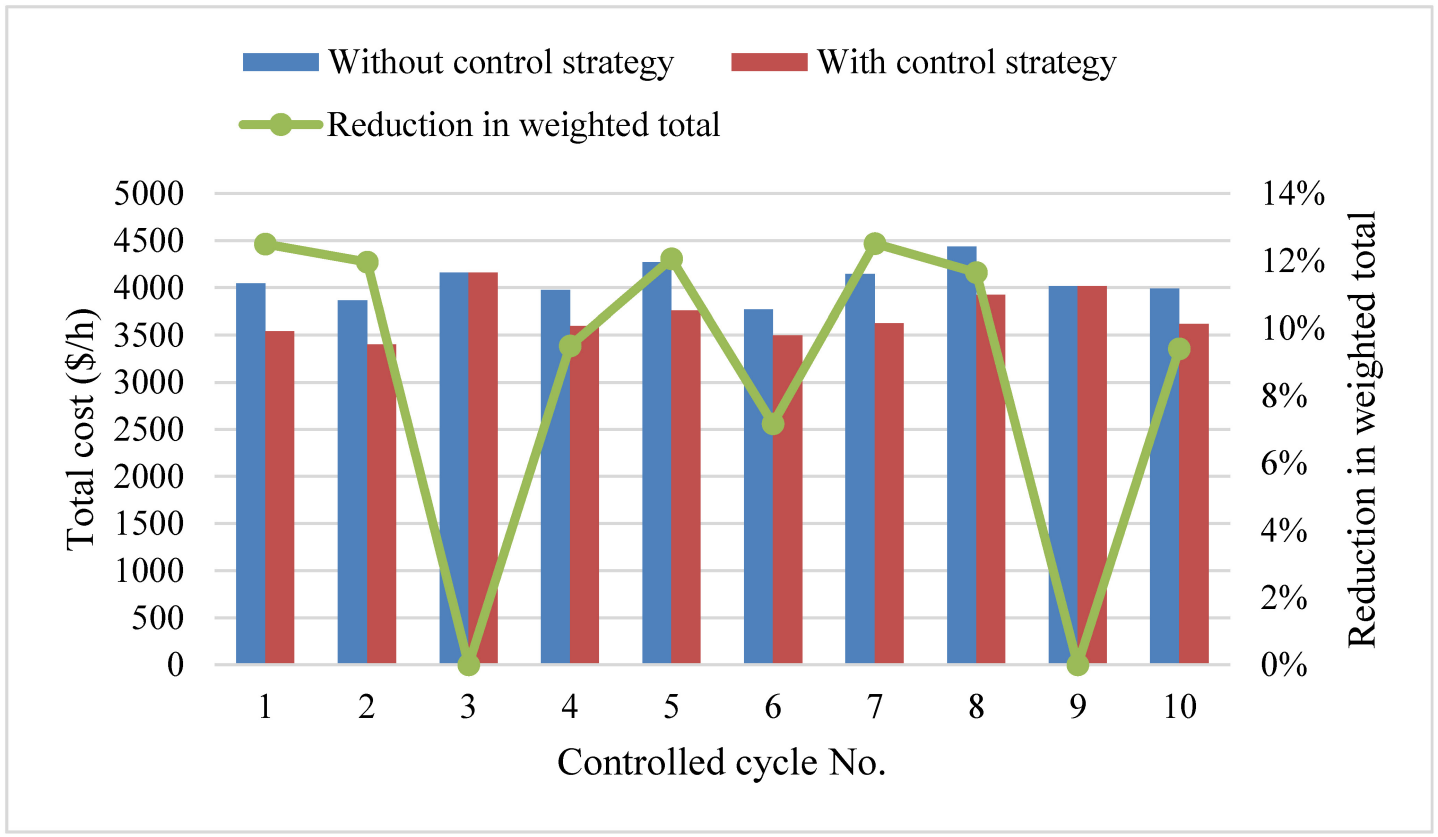
Total cost of historical data.

Furthermore, performances of traditional service and limited-stop service are compared. Under traditional service, buses stop at each station along the entire bus route and each *y*_*i*,*j*_ is set to 1. The design of a limited-stop service needs to adopt the above solution method, which searches *y*_*i*,*j*_, and then determines the proper skipping stations. The data used to test the effect of the traditional service and limited-stop service uses the same passenger demand and running time of buses between consecutive stations. The results of the total cost per hour in response to two services and the reduction in the total cost are shown in Fig 11.

Fig 11 shows that the limited-stop service has a strong effect on saving the total cost of the transit system with a high degree of unbalanced passenger demand. However, there are exceptions for data 3 and 9, where the degree of unbalanced passenger demand is 4.1 and 3.4, respectively, and where no proper skipping stations exist and the optimization scheme is stopping at entire stations. The reductions in total cost for data 3 and 9 are both zero. Besides the data 3 and 9, the limited-stop service can be implemented under a high degree of unbalanced passenger demand. Based on the tested data, there is at least a 7% reduction and the highest reduction is 13%. Therefore, the limited-stop service is not always valid for every pattern of passenger demand and it is necessary to determine the degree of unbalanced passenger demand to judge whether the limited-stop service should be implemented to decrease the total cost of the transit system.

## Conclusions

Our study is assuming an unbalanced distribution of passenger demand along the bus route, which most transit systems have to accommodate, and provides a limited-stop service for the entire bus fleet. To determine the condition of implementing the limited-stop service, it is useful to consider the degree of unbalanced passenger demand as a judgment condition.

In this study, the degree assessment of unbalanced passenger demand reaches the unbalanced distribution of passenger demand between stations and within the station. The variance of the unbalanced distribution of passenger demand is computed as the degree of unbalanced passenger demand. A high degree of unbalanced passenger demand indicates that the bus route needs to implement a limited-stop service. The design of the limited-stop service provides an optimization scheme that includes proper skipping stations for a bus fleet. The limited-stop service allows buses to skip several stations with low passenger demand and consecutive buses cannot skip the same station, considering the waiting time of passengers at those stations. As a result of the test, several conclusions can be formulated:

When comparing the total cost, including waiting time cost, in-vehicle time cost, and running time cost, between both with and without limited-stop service and under different degree of unbalanced passenger demand, changes in the total cost with limited-stop service are more obvious in response to different degrees of unbalanced passenger demand. The threshold should be set to 6.0. Under a low degree of unbalanced passenger demand, traditional service and implementing limited-stop service result in little difference. When the degree of unbalanced passenger demand is higher than the threshold, implementing limited-stop service can save the total cost of the transit system.

The design of a limited-stop service can respond well to a change in degree of unbalanced passenger demand under different distribution patterns. If applicable, a limited-stop service can save a total cost of about 10%.

Furthermore, there are several limitations of this study, which need to be addressed. The limited-stop service needs to be extended to branching corridors that are composed of several feeder lines and a trunk line. Real-time changes of passenger demand can also be integrated into the optimization. Other operational strategies, such as deadheading and short turning, could be combined to deal with the unbalance of passenger demand.

## Supporting information

S1 File Running time between stations of bus route number 6. (DOCX)
