# Supplementary material for: Design of limited-stop service based on the degree of unbalance of passenger demand
Source: PLoS One. 2018 Mar 5;13(3):e0193855. doi: 10.1371/journal.pone.0193855 (PMC5837195; doi:10.1371/journal.pone.0193855)
Supplement: S1 File — (DOCX) [file pone.0193855.s001.docx]

**S1 File. Running time between stations of bus route number 6.**

| Station No. | Running time between stations (min) |
| --- | --- |
| 1 | 0 |
| 2 | 0.299964 |
| 3 | 0.668822 |
| 4 | 1.586558 |
| 5 | 0.981482 |
| 6 | 1.756337 |
| 7 | 2.256594 |
| 8 | 2.509441 |
| 9 | 1.488094 |
| 10 | 1.351531 |
| 11 | 1.394087 |
| 12 | 1.817642 |
| 13 | 1.718505 |
| 14 | 1.806422 |
| 15 | 1.834472 |
| 16 | 1.141636 |
| 17 | 1.078083 |
| 18 | 1.460445 |
| 19 | 1.231397 |
| 20 | 1.618487 |
